# Examining the relationship between physical activity and sleep among university students

**DOI:** 10.3389/fspor.2025.1640770

**Published:** 2025-09-25

**Authors:** Nikoletta Barka, Feifei Wang, Robert Jarai, Szilvia Boros

**Affiliations:** ^1^ Doctoral School of Education, ELTE Eötvös Loránd University, Budapest, Hungary; ^2^ Sport Office, Budapest University of Economics and Business, Budapest, Hungary; ^3^ Faculty of Nursing, Medical College, Jiaxing University, Jiaxing, China; ^4^ Department of Psychology and Health Management, Faculty of Health and Sport Sciences, Széchenyi István University, Győr, Hungary

**Keywords:** physical activity, sitting time, sleep quality, university students, walking, WHO

## Abstract

**Objective:**

Physical activity and adequate sleep are essential for health and wellbeing. University students face distinct challenges affecting their habits. This study investigates sociodemographic impacts on physical activity and sleep patterns, and examines the association between physical activity and sleep quality in Hungarian university students.

**Methods:**

An online cross-sectional survey was conducted among students (*N* = 1,340, mean age 20.00 ± 1.59 years; 60.7% female and 39.3% male) from the Budapest University of Economics and Business. The survey was based on sociodemographic data, the Hunarian version of the International Physical Activity Questionnaire-Short Form (IPAQ-SF), and the Pittsburgh Sleep Quality Index (PSQI) questionnaires. In IPAQ-SF, respondents indicated physical activities lasting at least 10 min during the last seven days. Responses were categorised by WHO and IPAQ guidelines. Metabolic Equivalent of Task (MET) was calculated. Statistical analyses were conducted using IBM SPSS Statistics 29.0.0.0, with significance set at *p* < 0.05.

**Results:**

Most participants (85.8%–86.9%) performed below the WHO recommendations for moderate-intensity physical activity. Significant sex differences were noted in physical activity levels (*p* < 0.001 for vigorous intensity; *p* < 0.043 for moderate intensity), with men being more active than women. Regarding sleep quality, 57.1% of participants reported good sleep quality (PSQI 0–5), 36.1% had moderate sleep disturbances (PSQI 6–10), and 6.8% experienced poor to severe sleep disturbances (PSQI 11–21). Women reported significantly poorer sleep quality than men did (*p* < 0.001). Multiple linear regression analysis revealed a significant interaction between energy expenditure on physical activity and sports participation frequency (*β* = −0.09, *p* = 0.012), indicating that regular sports participation may buffer against potential negative effects of high overall physical activity on sleep quality. The model explained 3.1% of the variance in sleep quality (*R*^2^ = 0.031, *p* < 0.001).

**Conclusion:**

The relationship between physical activity (MET-minutes/week) and sleep quality was moderated by the frequency of sports participation. Given that poor sleep can negatively impact academic performance, health, and well-being, these findings support the promotion of organized sports within university settings. Interventions targeting both physical activity and sleep hygiene may yield synergistic benefits, particularly for students with sedentary lifestyles.

## 1 Introduction

Physical activity and sleep are fundamental components of health, with well-documented associations with physical, mental, and social well-being. According to the World Health Organization (WHO), adults should engage in at least 150 min of moderate-intensity or 75 min of vigorous-intensity physical activity weekly to maintain health (1). Insufficient sleep (less than 7 h per night) is linked to detrimental effects on human health. Physical inactivity and poor sleep are independently associated with adverse health outcomes, such as insulin resistance (2), cardiovascular disease (3), and immune system problems (4). In addition, lack of exercise is a major cause of chronic diseases (5). For example, participating at moderate intensity exercises had a decreased incidence of diabetes (6).

Exercises enhance sleep quality in multiple ways. Previous research has emphasised the importance of regular physical activity in improving sleep quality, with studies showing that physically active individuals report better sleep efficiency compared to their sedentary counterparts (7, 8). Robust findings demonstrate that people who are more physically active have a lower frequency of sleep disorders and better ratings of sleep quality (9, 10) Existing finding shows that adults who exercised for at least 30 min a day slept an average of 15 min longer than those who did not exercise (11) Conversely, poor sleep quality is associated with reduced motivation for physical activity, creating a bidirectional relationship that exacerbates health risks (12). Recent research demonstrated that exercise timing, intensity, and the presence of other zeitgebers may influence the association between physical activity and sleep quality (13).

College students face unique challenges today, including environmental context and resources (e.g., time constraints), social influences (e.g., exercising with others), and goals (e.g., prioritisation of physical activity), which may influence both physical activity patterns and sleep quality (14). Due to changes in lifestyle and behaviour, young adults are becoming particularly vulnerable to sleep deprivation and physical inactivity (15). Specifically, more than two-thirds of adolescents sleep less than eight hours per night in the United States (16), which is similar to findings among Jordanian university students (17). In China, less than 50% of students with sleeping troubles reported varying degrees of sleep disorders (18). Although sleep disorders differ internationally and locally, the global trend has been increasing in recent years. This study aimed to identify the association between physical activity and sleep quality by integrating sociodemographic factors among Hungarian university students.

### 1.1 Aim

The study aims to investigate the relationship between sleep and various components, including physical activity and demographic factors. Our objective is to assess the level of physical activity among students and determine whether physical activity and sleep are positively correlated.

Research questions:

1. What is the impact of sociodemographic factors on the physical activity and sleep patterns of Hungarian university students?
2. Is there an association between physical activity and sleep quality among university students?

## 2 Materials and methods

### 2.1 Participants

The present study recruited 1,340 students from the Budapest University of Economics and Business, selected in accordance with the institutional research ethics (licence number: 2022/388-2). Participation was open to all enrolled students, regardless of degree program or level, provided they consented to the study.

The mean age of participants was 20.00 ± 1.59 years (ranging from 18 to 31) included 526 males (39.3%) and 814 females (60.7%).

Before completing the questionnaire, an online privacy policy was provided, which they had to accept before they could complete the questionnaire.

### 2.2 Data capturing

Data collection was conducted using Microsoft Forms, which was distributed to all bachelor's students at the Budapest University of Economics via the Central Student Administration System in Hungary. The questionnaire survey targeted students enrolled in bachelor programs across all three faculties. It was completed online, anonymously, during the autumn semester of the 2022/2023 academic year, between September and December. A total of 1,340 students completed the questionnaire. Participation was entirely voluntary, and all students signed an online informed consent form prior to beginning the survey.

The survey consisted of three parts: (1) demographic questions (age, gender, marital status, place of residence, place of domicile, type of education, source of funding and whether student worked during their studies, (2) items related to university sports opportunities and time spent on various leisure activities (measured on a 0–6 Likert scale), and (3) two standardized questionnaires assessing physical activity and sleep quality.

Physical activity was measured using the Hungarian version of the Physical Activity Questionnaire-Short Form [IPAQ-SF; (19, 20)]. Respondents needed to indicate only physical activities that lasted at least 10 min during the last seven days. The International Physical Activity Questionnaire-Short Form (IPAQ-SF) is a standardized, self-report survey designed to assess physical activity levels in adults over the previous seven days. It collects information on the frequency and duration of vigorous, moderate, and walking activities, as well as time spent sitting, enabling researchers and health professionals to estimate overall physical activity and sedentary behavior.

Responses were categorised in multiple ways. One categorisation follows the World Health Organisation (WHO) recommendations for adults: moderate (150–300 min per week) and high (75–150 min per week) intensity physical activity (1).

Another categorisation was created according to the IPAQ-SF's own “Guidelines for Data Processing and Analysis of the International Physical Activity Questionnaire (IPAQ)-Short and Long Forms” (21). To calculate the IPAQ-SF Global score, the last 7-day period was used in addition to the above.

Based on the IPAQ Guidelines recommendation, the Metabolic Equivalent of Task (MET) was calculated as one of the measures of activity. MET values are multiples of the resting metabolic rate, and MET-minutes are calculated by multiplying the MET value of the activity by the minutes performed (21).

For measuring sleep, the Hungarian version of The Pittsburgh Sleep Quality Index (PSQI-HUN) was applied (22, 23). The PSQI contains 19 self-assessment questions and 5 additional questions. Only the 19 self-assessment questions count towards the PSQI global score. Questions were grouped into seven components, ranked from 0 to 3. When the scores of the seven components were added together, scores were obtained between 0 and 21, which were grouped into the following categories: Good Sleep Quality (0–5), Moderate Sleep Disturbance (6–10), Poor Sleep Quality (11–15), and Severe Sleep Disturbance (16–21).

### 2.3 Data processing

All collected data were initially cleaned and structured in Microsoft Excel before statistical analysis. Descriptive statistics (means, standard deviations, frequencies) were calculated to summarize the variables. To analyze associations between categorical variables, cross-tabulations were performed and assessed using Pearson's Chi-square test (*χ*^2^). For continuous outcomes, linear regression analyses were conducted to examine predictive relationships. The Shapiro–Wilk test was used to assess the normality assumption of the residuals in linear regression. All analyses were performed in IBM SPSS Statistics (Version 29.0.0.0), and the threshold for statistical significance was set at *α* = 0.05.

## 3 Results

### 3.1 Sociodemographic data

In terms of marital status, more than half were single (54.8%), 42.0% were in a relationship, 0.8% were married, and 2.4% indicated other categories, with some providing free-form answers such as “I am in an open relationship”.

When asked where they live most of the time, 56.1% of respondents indicated the capital, 11.2% a county town or a large city, 20.3% a small town, 5.5% a village, 4.3% a township, 2.4% a large municipality, and 0.1% a farm. However, it should be noted that all university classes are held in the capital.

Most respondents live on their own property (41.7%), 29.6% in rented accommodation, 10.1% in a dormitory, and 18.6% in other accommodation.

Most respondents (99.7%) study full-time, with 58.3% studying state-funded and 41.7% self-financed. More than half of the respondents (51.0%) work while studying at university (Table 1).

**Table 1:** The relationship between physical activity level and timing and sleep quality and hygiene in healthy individuals: a cross-sectional study.

*N* = 1,340							
Sex	814 (60.7%) Women	526 (39.3%) Men					
Marital status	Single (54.8%)	In a relationship (42.0%)	Married (0.8%)	Other (2.4%)			
Place of Residence	Capital city (56.1%)	County town or large city (11.2%)	Small town (20.3%)	Village (5.5%)	Township (4.3%)	Large municipality (2.4%)	Farm (0.1%)
Type of Residence	Own property (41.7%)	Rented accommodation (29.6%)	Dormitory (10.1%)	Other (18.6%)			
Mode of study	Full-time (99.7%)	Correspondence (0.3%)					
Source of funding	State-funded (58.3%)	Self-funded (41.7%)					
Work while studying	Yes (51.0%)	No (49.0%)					

#### 3.1.1 Comparison of demographic factors and physical activity

Physical activity was examined in relation to several factors (e.g., sociodemographic data, time spent sitting, walking, etc.).

For the seven days before completing the questionnaire, the mean time spent sitting was 307.67 min per day, with a standard deviation of 237.61, and the median was 270 min. Typically, though not necessarily in the 7 days before the completion, the results changed. In this case, the mean was 300.31 min per day, with a standard deviation of 213, while the median was 255.

The mean time spent walking in the “7 days” before completion was 250.96 min, with a standard deviation of 443.22, and the median was 120. In contrast, they “typically” walked for 215.22 min, with a standard deviation of 402.41 and a median of 100.

A significant relationship was found between moderate physical activity in the “7 days” before completion and sitting (*p* < 0.003) and walking (*p* < 0.001).

At the same time, a significant relationship (*p* < 0.001) was observed for both sitting and walking for “typically” performed moderate physical activity.

On comparing sitting and walking with vigorous-intensity physical activity for the “7 days” before completion, no significant correlation was found (sitting-*p* < 0.261 and walking-*p* < 0.202).

For “typically” vigorous-intensity physical activity, a significant relationship was found with sitting (*p* < 0.009) and walking (*p* < 0.001).

The responses of the study participants to moderate and vigorous-intensity physical activity were compared with the WHO recommendations for moderate (150–300 min/week) and vigorous (75–150 min/week) intensity (Table 2).

**Table 2:** WHO distribution of moderate and vigorous-intensity physical activity in the past seven days and typically.

Physical activity intensity	Below WHO recommendation	According to WHO recommendation	Above WHO recommendation	Total
Moderate-intensity physical activity (in the past seven days)	1,122 (85.8%)	128 (9.8%)	58 (4.4%)	1,308
Vigorous-intensity physical activity (in the past seven days)	788 (59.9%)	321 (24.4%)	207 (15.7%)	1,316
Moderate-intensity physical activity (typically)	1,132 (86.9%)	121 (9.3%)	50 (3.8%)	1,340
Vigorous-intensity physical activity (typically)	754 (57.4%)	340 (25.9%)	219 (16.7%)	1,313

As not all respondents answered all questions, we do not have 1,340 observations in each row.

The majority of respondents (85.8% and 86.9%) engaged in less moderate-intensity physical activity than recommended by the WHO while 9.8% and 9.3% met the recommendations, and 4.4% and 3.8% exceeded them. Of the respondents, 59.9% and 57.4% did less than the WHO-recommended vigorous-intensity physical activity, 24.4% and 25.9% met the recommendations, and 15.7% and 16.7% did more.

Respondents preferred moderate physical activity. They were more likely to engage in vigorous-intensity physical activity at or above the WHO recommendation than at moderate intensity.

Physical activity was examined in relation to several factors, including sex, marital status, and working while studying.

Differences were observed between physical activity in regard to sex. For moderate physical activity, most of both sexes (considering the last “7 days” and “typically”) performed less moderate-intensity physical activity than the WHO recommendations—women 87.6% and 88.3%, men 82.9% and 84.7%, respectively. Additionally, men were more active, in percentage terms, within and above the recommended range in both categories (within seven days and typically) (Table 3).

**Table 3:** WHO distribution of moderate and vigorous-intensity physical activity and IPAQ-SF categories and relationship by sex.

Physical activity intensity		Overall	Women	Men	*p*-Value
Moderate-intensity physical activity (in the past seven days)	Below	1,122 (85.8%)	694 (87.6%)	428 (82.9%)	***p*** **<** **0.043**
	According	128 (9.8%)	70 (8.8%)	58 (11.2%)	
	Above	58 (4.4%)	28 (3.5%)	30 (5.8%)	
Moderate-intensity physical activity (typically)	Below	1,132 (86.2%)	696 (88.3%)	436 (84.7%)	*p* < 0.097
	According	121 (9.3%)	68 (8.6%)	53 (10.3%)	
	Above	50 (3.8%)	24 (3.0%)	26 (5.0%)	
Vigorous-intensity physical activity (in the past seven days)	Below	788 (59.9%)	554 (69.5%)	234 (45.1%)	***p*** **<** **0.001**
	According	321 (24.4%)	163 (20.5%)	158 (30.4%)	
	Above	207 (15.7%)	80 (10%)	127 (24.5%)	
Vigorous-intensity physical activity (typically)	Below	754 (57.4%)	528 (66.2%)	226 (43.8%)	***p*** **<** **0.001**
	According	340 (25.9%)	178 (22.3%)	162 (31.4%)	
	Above	219 (16.7%)	91 (11.4%)	128 (24.8%)	
IPAQ-SF	Low	958 (71.5%)	632 (77.6%)	326 (62.0%)	***p*** **<** **0.001**
	Moderate	311 (23.2%)	144 (17.7%)	167 (31.7%)	
	High	71 (5.3%)	38 (4.7%)	33 (6.3%)	

Bold values are significant at the 5% level.

Although there are no major differences between the moderate-intensity categories, there is still a significant difference between the information provided in the last “7 days” and the sexes (*p* < 0.043). The same applies for typically conducted exercises (*p* < 0.097).

A significant (*p* < 0.001) difference was observed between vigorous-intensity physical activity and sex, both for the “last 7 days” and the “typically” categories. In both the WHO-recommended (30.4% and 31.4%) and above (24.5% and 24.8%) categories, men were more likely to engage in vigorous-intensity physical activity.

In addition to the WHO categories, we also examined the IPAQ categories (Low, Medium, and High). One of the criteria for this is also the MET value for walking, moderate, and vigorous physical intensity.

Looking at the IPAQ-SF categories, we found that most respondents, 958 (71.5%), belonged to Category 1 (Low) physical activity, 311 persons (23.2%) were classified in Category 2 (Moderate), and 71 persons (5.3%) in Category 3 (High).

In terms of MET values, which included walking, moderate, and vigorous-intensity physical activity according to the IPAQ-SF scoring system, respondents reported a mean of 670.3 MET/week. The standard deviation was 1,167.2, while the median was 140.0.

A large proportion of students were in the low category, with 77.6% of women and 62.0% of men. When comparing the moderate and high categories, both women and men were more likely to be in the moderate category (17.7% of women and 31.7% of men). A significant correlation (*p* < 0.001) was found.

Table 4 compares physical activity categories with age groups, in which a significant correlation was also found (*p* < 0.046). According to the IPAQ-SF, 22.0% of 18-year-olds engage in moderate-intensity physical activity and 3.7% in high-intensity physical activity. For 19–20-year-olds, the rates are 22.6% and 4.4% respectively, and for those aged 21 and above, the rates are 25.2% and 7.9%.

**Table 4:** Relationship between IPAQ-SF categories and sex (*p* < 0.046).

Age	IPAQ-SF Low	IPAQ-SF Moderate	IPAQ-SF High	Total
≤18	159 (74.3%)	47 (22.0%)	8 (3.7%)	214 (100%)
19–20	534 (73.1%)	165 (22.6%)	32 (4.4%)	731 (100%)
≥21	263 (66.9%)	99 (25.2%)	31 (7.9%)	393 (100%)

No significant difference was found between marital status (*p* < 0.432), place of domicile (*p* < 0.451), place of residence (*p* < 0.332), form of education (*p* < 0.449), and form of financing (*p* < 0.117), but a significant difference was found with working while studying (*p* < 0.0012).

### 3.2 Comparison of sleep and other parameters

In terms of sleep, respondents required a mean of 22.24 min to fall asleep. Most people, 672 (50.1%), described their sleep in general as “less good”, 538 (40.1%) as “very good”, 97 (7.2%) as “poor”, and 31 (2.3%) as “very poor”.

Of the respondents, 1,044 (78%) do not take medication to help them sleep, 136 (10.2%) take medication less than once a week, 73 (5.5%) once or twice a week, and 85 (6.4%) three or more times a week. The question covered both prescription and over-the-counter medicines.

There were significant differences between genders in sleep characteristics (*p* < 0.013), taking medication (*p* < 0.001), staying awake (*p* < 0.001), and maintaining interest (*p* < 0.001). Looking at the PSQI Global scores, 717 respondents (57.1%) were classified as having “Good Sleep Quality”. Of the respondents, 452 (36.1%) experienced “Moderate Sleep Disturbance”, 82 (6.5%) had “Poor Sleep Quality”, and 4 (0.3%) had “Severe Sleep Disturbance”.

The relationship between PSQI global score and sex (*p* < 0.001) was significant. However, no such correlation was found between marital status (*p* < 0.444), place of domicile (*p* < 0.950), place of residence (*p* < 0.144), form of financing (*p* < 0.965), form of education (*p* < 0.681), and work while studying (*p* < 0.792). The distribution of PSQI Global categories and sex is shown in Table 5.

**Table 5:** Relationship between the PSQI global categories and sex.

Gender	Good sleep quality (0–5)	Moderate Sleep Disturbance (6–10)	Poor Sleep Quality (11–15)	Severe Sleep Disturbance (16–21)	Total
Females	352 (46.9%)	331 (44.1%)	64 (7.5%)	4 (0.5%)	751 (100%)
Males	293 (60.9%)	166 (34.5%)	22 (4.5%)	0 (0.0%)	481 (100%)

When comparing the PSQI Global score and age, no significant correlation was found. For distribution data, see Table 6.

**Table 6:** Sleep quality (global PSQI) based on age.

Global PSQI	Age categories (years)				
	Total	18	19–20	≥21	*p*-Value
Good sleep quality (0–5)	645 (52.4%)	107 (52.9%)	358 (53.4%)	180 (50.1%)	*p* < 0.859
Moderate Sleep Disturbance (6–10)	497 (40.3%)	83 (41.1%)	266 (39.6%)	148 (41.2%)	
Poor Sleep Quality (11–15)	86 (7.0%)	11 (5.4%)	44 (6.5%)	31 (8.6%)	
Severe Sleep Disturbance (16–21)	4 (0.3%)	1 (0.5%)	3 (0.4%)	0 (0.0%)	

No significant correlation was found between minutes spent sitting and walking in the last seven days, typically relative to each other, and the number of hours of sleep.

A significant difference was found between PSQI Global and time spent sitting in the period immediately preceding completion (*p* < 0.016), while no significant difference was observed in the typical category (*p* < 0.295).

When comparing physical activity according to WHO categories with the global score of the PSQI, no significant correlation was found, nor with the combined score of the IPAQ (*p* < 0.132) (Table 7).

**Table 7:** Association between IPAQ-SF categories and PSQI global categories (*p* < 0.132).

IPAQ-SF categories	Good sleep quality (0–5)	Moderate sleep disturbance (6–10)	Poor sleep quality (11–15)	Severe sleep disturbance (16–21)	Total
Low	501 (55.5%)	342 (37.9%)	56 (6.2%)	3 (0.3%)	902
Moderate	176 (61.1%)	89 (30.9%)	23 (8.0%)	0 (0.0%)	288
High	40 (60.6%)	22 (33.3%)	3 (4.5%)	1 (1.5%)	66
Total	717 (57.1%)	453 (36.1%)	82 (6.5%)	4 (0.3%)	1,256

### 3.3 Results of linear regression analysis: physical activity and sleep quality

#### 3.3.1 Descriptive statistics

The sample consisted of 1,232 participants with a mean age of 19.93 years (SD = 1.60). The sample included both males (39%) and females (61%). Participants reported an average-physical-activity energy expenditure of 1.67 thousand MET-minutes per week (SD = 1.48) and a mean sports participation frequency of 2.08 on a 0–6 Likert scale (SD = 1.52). The average global PSQI score was 5.76 (SD = 3.03), indicating generally poor sleep quality in the sample (scores >5 indicate poor sleep quality).

#### 3.3.2 Linear regression analysis

A multiple linear regression analysis was conducted to examine the relationship between physical-activity energy expenditure (MET-minutes/week) and sleep quality (PSQI global score), while controlling for gender, age, and sports participation frequency. An interaction term between MET-minutes and sports participation was included to investigate whether the effect of physical activity energy expenditure on sleep quality varies depending on the frequency of sports participation.

The regression model was statistically significant, *F* (5, 1,226) = 7.96, *p* < .001, but explained only a small proportion of the variance in sleep quality (*R*^2^ = 0.031, adjusted *R*^2^ = 0.028). The results revealed a significant main effect of physical activity energy expenditure (*β* = 0.24, *p* = .012), indicating that higher MET-minutes per week were associated with poorer sleep quality when sports participation was zero. Sex was also a significant predictor (*β* = 0.98, *p* < .001), with females reporting poorer sleep quality than males did. Age was not significantly associated with sleep quality (*β* = 0.001, *p* = .988).

Importantly, a significant interaction was found between physical-activity energy expenditure and sports participation frequency (*β* = −0.09, *p* = .012). This interaction suggests that the relationship between physical-activity energy expenditure and sleep quality is moderated by the frequency of sports participation. Specifically, as sports participation frequency increases, the positive relationship between MET-minutes and poor sleep quality diminishes. This finding supports the rationale for including the interaction term, as it reveals that the effect of physical activity on sleep quality is not uniform across different levels of sports participation.

Physical activity is any bodily movement produced by skeletal muscles, which expends energy, including a wide range of activities such as walking, gardening, household chores, and occupational tasks (24–26). In contrast, sports is a subset of physical activity that is typically organised, often competitive, and involves specific rules or goals, either individually or as part of a team (27, 28). While all sports is physical activity, not all physical activity qualifies as sports. Sports often brings additional psychosocial and personal development benefits but may also carry risks such as injury or burnout (27).

**Figure 1:**
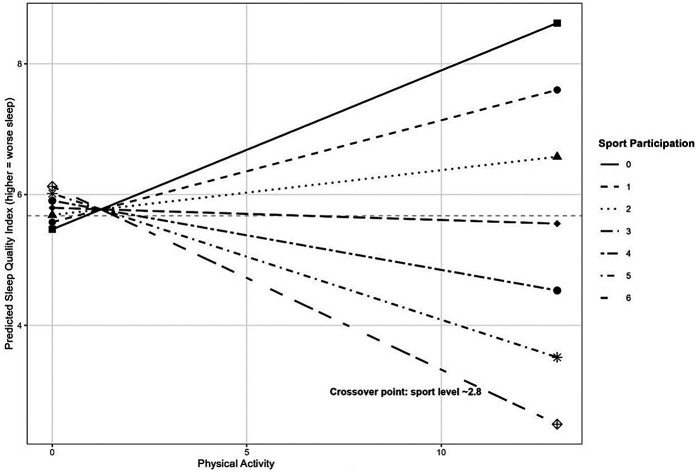
Effect of physical activity on sleep quality by sports participation level.

Respondents indicated how many hours of physical activity they engage in an average per week, based on their answers to the questionnaire. Their responses were recorded on a scale of 0–6 (0 = none, 1 = 1–2 h, 2 = 2–4 h, 3 = 4–6 h, 4 = 6–8 h, 5 = 8–10 h, 6 = more than 10 h). The results were compared with their PSQI Global score. The results show that individuals who move more, sleep better (Figure 1).

##### 3.3.2.1 Rationale for including the interaction effect

The interaction between physical-activity energy expenditure and sports participation was included in the model based on theoretical considerations that different types of physical activity might have differential effects on sleep quality. While overall physical activity (measured by MET-minutes) includes various forms of daily activities that might be associated with increased arousal or stress, structured sports participation might represent more regulated physical activity with potential sleep-enhancing benefits.

The significant interaction in our analysis confirms this rationale, suggesting that regular sports participation may buffer against the potential negative effects of high overall physical activity on sleep quality. This finding highlights the importance of considering not just the quantity of physical activity (MET-minutes) but also its context and structure (sports participation) when examining relationships with sleep outcomes.

##### 3.3.2.2 Residual analysis and model Fit

The Shapiro–Wilk test indicated that the residuals of the regression model deviated from normality (*W* = 0.97, *p* < .001). However, as noted by Ghasemi and Zahediasl (29), with large sample sizes (*N* > 30), violating the normality assumption should not cause major problems, and parametric procedures can be used even when the data are not normally distributed. In our case, with a substantial sample size of 1,232 participants, the Central Limit Theorem suggests that the sampling distribution of the means would approach normality regardless of the underlying distribution (30). Residual plots (not shown) were also visually inspected to assess homoscedasticity and linearity assumptions.

The model explained only a small proportion of the variance in sleep quality (*R*^2^ = .031, adjusted *R*^2^ = .028). While statistically significant, this low R-squared value indicates that factors beyond those included in our model likely contribute substantially to variations in sleep quality. This finding is consistent with the complex, multifactorial nature of sleep quality as described by Buysse (31), who emphasised that sleep is influenced by numerous physiological, psychological, environmental, and behavioural factors. Previous research by Kredlow et al. (32) in their meta-analysis of physical activity and sleep also found modest effect sizes, suggesting that while physical activity contributes to sleep quality, it represents just one of many influential factors. The significant predictors and interaction identified in our model, despite the low overall variance explained, still provide valuable insights into the relationship between specific aspects of physical activity and sleep quality in this population.

## 4 Discussion

The study aimed to explore the relationship between physical activity, sitting, walking, and sleep quality. In examining the differences between moderate and vigorous physical activity as well as the time spent sitting, several studies have found that while both intensities mitigate the risks associated with sitting, vigorous activity provides more efficient protection per time unit (33, 34). Keadle et al. and Stamatakis et al. argued that replacing sedentary time with vigorous activity leads to more substantial cardiovascular and metabolic benefits than moderate-intensity activity of the same duration. It is important to note, however, that several studies linked these associations to health and behavioural outcomes rather than examining the two components independently (34–37). Similarly, our study identified differences between moderate and vigorous physical activity and sitting, but no health analyses were conducted.

The same applies to studies examining the relationship between the intensity of physical activity and walking, which mainly looked at health risks. Across the studies, greater overall physical activity—especially at moderate to vigorous intensities—was associated with larger reductions in health risks such as type 2 diabetes, metabolic syndrome, and poorer cognitive or mental health, though walking even at lower intensities still provided some significant benefits. While moderate-to-vigorous physical activity offered the most pronounced improvements, brisk walking or high volumes of walking similarly reduced certain health risks, indicating that both walking and higher-intensity activity are effective, with intensity and duration enhancing the benefits (38–41). In another large Australian cohort study, individuals who engaged in moderate physical activity as well as higher levels of vigorous physical activity had significantly lower all-cause mortality rates, supporting the inclusion of vigorous exercise in public health recommendations (42). Our study also found differences between moderate and vigorous physical activity and walking, but no health analyses were connected.

Expanding this discussion, it is widely recognized in the literature that vigorous physical activity tends to confer greater cardiometabolic benefits and mortality risk reductions per time unit than moderate activity. For instance (43) conducted a systematic review and harmonised meta-analysis of accelerometer-assessed physical activity, sedentary time, and their associations with all-cause mortality across eight prospective cohort studies including over 36,000 adults. Results showing that higher total physical activity levels, regardless of intensity, are associated with substantially lower risk of all-cause mortality in a nonlinear dose-response pattern. Specifically, even light physical activity reduced mortality risk significantly, while greater sedentary time was linked to increased mortality risk. These findings highlight that increasing physical activity and reducing sedentary behavior are important strategies to prevent premature death in this population. Furthermore, Saunders et al. (44), emphasized with the overview of eighteen systematic reviews that high levels of sedentary behavior are consistently associated with negative health outcomes in adults, including poorer cognitive function, increased depression, reduced physical function, and lower quality of life. Additionally, breaking up prolonged sitting and reducing total sedentary time may improve body composition and cardiometabolic risk markers, although evidence quality varies from low to high across studies. Interestingly, while most sedentary behaviors correlate negatively with health, computer and internet use may have a positive association with cognitive function in adults.Gender differences exist in physical activities and age IPAQ-SF physical activity categories. Men are generally more likely to report high activity levels, particularly for vigorous and moderate intensities, though some studies in older adults show women may report higher total activity (45–47). Our research confirms the former finding. One comprehensive global review from (48) discusses gender disparities in physical activity levels and types, highlighting men's greater participation in vigorous physical activities and women's generally higher engagement in light-intensity domestic activities, which may be under-reported by common questionnaires. Several IPAQ-SF studies of university students have shown that the majority of students fall into the moderate-to-high physical activity category, with a minority in the low category (49, 50). In our research, however, most respondents were in the “low” category, as found in Kurçer et al.'s (51) research. While some studies indicate a majority in moderate or high physical activity categories, others reveal a considerable proportion—sometimes even a majority—falling into the low activity group. In a large multicountry study involving 23 low-, middle-, and high-income countries, the prevalence of physical inactivity among university students was 41.4%, with national rates ranging from 21.9% up to 80.6%. Notably, in several contexts, more than half of students did not achieve recommended levels of physical activity, particularly in specific regions or countries (52). A systematic review covering 19 studies from 27 countries showed that more than half of university students in the United States and Canada are not sufficiently active to obtain health benefits; this trend is paralleled internationally, especially among women and students living on campus (53). Across the typical university age range (roughly 18–25 years), there is little evidence of significant differences in IPAQ-SF activity levels by age. Most studies do not report strong age-related trends within this relatively homogeneous group (50, 51, 54). Contrary to previous evidence, our data shows a significant relationship between age and IPAQ-SF intensity categories. Older participants are more likely to be in moderate and high IPAQ-SF categories.

Multiple studies have examined the relationship between the PSQI and time spent sitting, consistently finding that greater sedentary time is associated with poorer sleep quality (55–58). With our sample size, there is a significant correlation between PSQI Global and sitting time in the past seven days, but its value is practically negligible. This aligns with findings from Kredlow et al. (32), who conducted a meta-analytic review highlighting that sedentary behavior negatively correlates with sleep quality but with small effect sizes. Therefore, the correlation observed in our study is consistent with these nuanced findings suggesting multiple interacting factors influence sleep beyond sedentary time alone.

There is consistent evidence that higher levels of physical activity and regular sports participation are associated with better sleep quality, as measured by lower PSQI global scores (54, 59–61). Our results also indicate that individuals who move more sleep better. The four studies share a common finding that higher levels of physical activity are associated with better sleep quality among university or healthcare students. They consistently report that individuals who engage more in physical activity experience improvements in subjective sleep measures such as sleep quality, sleep hygiene, or sleep disturbances. Additionally, these studies highlight the importance of contextual or moderating factors—including pandemic conditions, self-control, and knowledge about sleep hygiene—that can influence the strength or nature of the relationship between physical activity and sleep quality. Overall, they underscore physical activity as an important positive correlate or predictor of improved sleep outcomes in young adult student populations.

It is important to note that the benefits of physical activity go far beyond improving sleep, as they have numerous positive effects on physical, mental, and emotional health. It is important to emphasize that it is never too late to develop a love of physical activity (even during university years) and that the key to lasting commitment lies not so much in the specific type of exercise, but rather in finding enjoyable activities that promote overall well-being. Several studies have shown that exercise has measurable health benefits for university students. One study found that yoga exercise intervention notably improved comprehensive well-being and various dimensions of subjective and psychological well-being among female college students. The yoga group showed significant increases in life satisfaction and altruistic behavior (62). Another research with university students who participated in Muay Thai exercises for six weeks experienced significant improvements in quality of life, self-control, and psychological well-being compared to the control group (63). A 15-week structured swimming program improved college students' mental health, emotional regulation, social connections, and coping mechanisms for academic stress through gradually increased intensity and training complexity (64).

### 4.1 Strengths, limitations, and future research

A major strength of the present study lies in its large sample size (1,340 participants), which enhances the reliability of the findings. Furthermore, the study's comprehensive approach to assessing physical activity—including the application of both WHO recommendations and IPAQ scoring—allows for a more nuanced analysis when compared alongside standardized measures of sleep, specifically the PSQI. This multifaceted methodology contributes to the robustness of the study's results.

Nevertheless, several limitations must be acknowledged. The participant pool consisted predominantly of full-time undergraduate students attending the Budapest University of Economics and Business, which restricts the generalizability of the findings to broader student populations. In order to more thoroughly explore the association between physical activity and sleep, future research should seek to recruit a more diverse sample, including part-time students, those engaged in various work schedules, and individuals from differing academic institutions. Moreover, the study's questionnaire focused primarily on sociodemographic information, sedentary behavior, and physical activity, thereby omitting other potentially influential variables such as dietary patterns, mental health status, technology use, or perceived stress levels. The inclusion of these factors in subsequent studies could provide deeper insight into the determinants of sleep quality.

It should also be noted that the use of self-reported, quantitative questionnaires introduces certain methodological constraints. Self-report measures are susceptible to bias, such as inaccurate recall or social desirability, which may affect data validity. Additionally, relying exclusively on quantitative data limits the ability to uncover more complex or subjective aspects of the relationship between physical activity and sleep. Incorporating qualitative methods, such as in-depth interviews or focus group discussions, could yield richer, more contextualized information and facilitate a deeper understanding of the observed associations.

For these reasons, future research should aim to include a more heterogeneous sample, consider a broader range of influencing factors, and utilize both quantitative and qualitative methodologies. Such approaches would contribute to a more comprehensive and generalizable understanding of the factors impacting sleep quality among university students.

## 5 Conclusion

This research offers a novel contribution by examining the sleep and physical activity habits of a large sample of Hungarian university students (*N* = 1,340), with attention to key sociodemographic variables. Although the statistical model explained a modest proportion of the variance in sleep quality (adjusted *R*^2^ = 0.028), the findings revealed a meaningful interaction: the relationship between physical activity (MET-minutes/week) and sleep quality was moderated by the frequency of sports participation. Specifically, higher physical activity levels were associated with poorer sleep quality only when no sports were performed—suggesting that structured physical activity may buffer against the negative effects of high overall energy expenditure on sleep.

These insights highlight the need to distinguish between general physical activity and regular sports engagement. Given that poor sleep can negatively impact academic performance, health, and well-being, these findings support the promotion of organised sports within university settings. Interventions targeting both physical activity and sleep hygiene may yield synergistic benefits, particularly for students with sedentary lifestyles.

## Data Availability

The datasets presented in this article are not readily available because The ethics licence and the privacy policy require the data to be stored securely on a trusted internal computer. The data can be accessed by the data owner after token identification. Requests to access the datasets should be directed to The Research Ethics Committee, keb@ppk.elte.hu.
